# Quantifying Ligand‐to‐Protein Distances in Complex Environments Using Intermolecular ^19^F PRE NMR Spectroscopy

**DOI:** 10.1002/cbic.70309

**Published:** 2026-04-17

**Authors:** Yannick Werle, Martha‐Louise Inderfurth, Christopher J. Lang, Michael Kovermann

**Affiliations:** ^1^ Department of Chemistry Universität Konstanz Konstanz Germany; ^2^ Graduate School of Chemical‐Biology (KoRS‐CB) Universität Konstanz Konstanz Germany

**Keywords:** cell lysate, fluorine, molecular crowding, nuclear magnetic resonance spectroscopy, PRE

## Abstract

The determination of structural features is crucial to understand the interplay between structure and function of biomolecules and biomolecular complexes. In this context, nuclear magnetic resonance (NMR) spectroscopy provides experimental approaches, one of which is paramagnetic relaxation enhancement (PRE). Thus, placing a paramagnetic center and fluorine at strategic sites within (bio)molecules forming a complex enables the determination of distances through a straightforward, one‐dimensionally guided NMR spectroscopic setup. Moreover, the almost absence of fluorine in biomolecules found in nature allows performing experimental work using cell‐like or *in cell* conditions. Here, we made use of a single‐cysteine mutant of *Bacillus subtilis* cold shock protein B (*Bs*CspB) equipped with a paramagnetic spin label in complex with a fluorine‐labeled variant of singly stranded DNA ligand dT4 to acquire intermolecular, ^19^F‐based PREs. The distance between *Bs*CspB and fluorine in dT4 has then been probed using three different experimental settings: in vitro, molecular crowding, and cell lysate conditions. Our data suggests that the intermolecular distance between the paramagnetically spin‐labeled protein and the fluorine‐labeled ligand does not change significantly using the three different conditions. This matches results regarding the conservation of binding affinities determined for this biomolecular complex using the three different conditions.

## Graphical abstract

Paramagnetic relaxation enhancement (PRE) represents a valuable tool in nuclear magnetic resonance spectroscopy to characterize dynamic and structural features of (bio)molecules. Here, intermolecular ^19^F PREs have been acquired between a spin‐labeled protein and fluorinated singly stranded DNA using different experimental conditions. The results indicate that the ligand‐to‐protein distance remains conserved when diluted, crowded, and cell lysate conditions are compared.

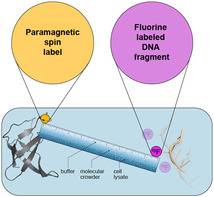

## 1 Introduction

One of the most prominent interactions existing in the field of biology is that between proteins and DNA molecules. For example, replication of DNA necessitates the concerted action of numerous specific proteins and enzymes, which are involved in all stages of this process [1]. In these types of interactions, the particular structure formed is strongly related to functionality, making it a subject of high relevance for researchers working in this field [2, 3]. Artificial intelligence (AI) has gained prominence in recent years, with the capacity to predict the structure of biomolecules and even biomolecular complexes [4]. However, despite the substantial improvements in the reliability in predicting the three‐dimensional structure of particular proteins, AI faces limitations in predicting the three‐dimensional structure of biomolecular complexes [5, 6, 7]. Consequently, the structural parameters predicted by AI still require validation through experimental data, provided by, e.g., high‐resolution nuclear magnetic resonance (NMR) spectroscopy. Moreover, the limitations of AI regarding structure prediction become also evident when specific experimental conditions, such as (macro)molecular crowding, are chosen which may impact structural features of biomolecules and biomolecular complexes that are studied [8].

The spatial distance *d* between two specified sites represents a direct structural restraint characterizing biomolecules and biomolecular complexes. Advantageously, NMR spectroscopy offers two primary experimental approaches to precisely probe this distance: the nuclear Overhauser effect (NOE) and the paramagnetic relaxation enhancement (PRE). Whereas the NOE is restricted to rather short distances (*d* ≤ 6 Å for proton–proton interactions [9]), PREs have been shown to be sensitive to rather large distances (*d* ≤ 35 Å) [10, 11].

PREs rely on the feature of paramagnetic species, such as radicals or certain metals, to enhance the longitudinal and/or transverse relaxation rate constants of nuclei through dipolar interactions in a distance‐dependent manner. Since paramagnetic moieties may not be inherently present in biomolecules, they have to be added artificially. The most abundantly used spin labels in PRE NMR spectroscopic experiments are nitroxide‐based radicals, such as *S*‐(1‐oxyl‐2,2,5,5‐tetramethyl‐2,5‐dihydro‐1H‐pyrrol‐3‐yl)methyl methanesulfonothioate (MTSL) or 3‐maleimido‐2,2,5,5‐tetramethyl‐1‐pyrrolidinyloxy (3MPrx) [12, 13], which can be attached to proteins via solvent‐exposed cysteine residues in a site‐specific manner [14, 15]. Originally, PRE NMR spectroscopic experiments have been performed by monitoring and analyzing resonance signals originating from ^1^H nuclei [16].

However, the ^19^F nucleus—besides using ^1^H nuclei—provides researchers with an attractive approach to study structural properties of biomolecules and biomolecular complexes based on the PRE methodology. This is attributed to the inherent properties of fluorine, comprising the natural abundance of 100%, the high gyromagnetic ratio, the high sensitivity to changes in its surroundings, and a broad range of the chemical shift scale [17, 18]. Advantageously, it is almost completely absent in naturally occurring biomolecules, enabling to conduct experimental work in more intricate settings, such as (macro)molecular crowding or cell lysate conditions [19, 20]. As an intermediate setting between diluted and cell‐like conditions, (macro)molecular crowders can also be utilized to model the intracellular scenario. The most used crowding agents include polyethylene glycol or dextran of different molecular weight [21].

Consequently, ^19^F PRE NMR studies have been successfully conducted to probe biomolecular properties, while primarily applying diluted (in vitro) conditions. It was, e.g., specifically demonstrated that ^19^F and ^1^H_N_ PREs observed for the protein CV‐N agree well with each other [22]. Moreover, ^19^F‐based PREs have been used, e.g., to study conformational changes of an intrinsically disordered protein [23] or to determine intersubunit distances of a pentameric ion channel [24]. It has also been demonstrated that ^19^F‐based PREs can be reliably determined inside mammalian cells [25], highlighting a broad potential for various biological applications.

Here, we present a quantitative approach that employs ^19^F NMR PREs aiming to determine intermolecular distances characterizing a protein–DNA complex. The experimental design includes different settings enabling to distinguish between pure in vitro and cell‐like conditions. The biomolecular complex probed here comprises two differently spin‐labeled variants of the *Bacillus subtilis* cold shock protein B (*Bs*CspB, *M*_W_ = 7.3 kDa) and a fluorine‐labeled derivative of the ligand molecule tetrathymidine (4FdT4, *M*_W_ = 1.2 kDa). The work outlined here is motivated by the observation that the binding affinity (in terms of the *K*_D_ value) of tetrathymidine (dT4) and *Bs*CspB does not change significantly when in vitro and cell‐like conditions are compared [26, 27]. However, the binding affinity does not report on the intermolecular distance(s) between the two interacting molecules.

Consequently, a singly cysteine‐containing variant of *Bs*CspB, S11C‐*Bs*CspB, has been prepared and equipped with different PRE spin labels. The transverse ^19^F PRE enhancement rate Γ_2_ of fluorine‐labeled dT4 has then been determined experimentally, in buffer (20 mM Na_2_HPO_4_ pH = 7.0, named “diluted conditions”), in the presence of the macromolecular crowding agent dextran 20 (*c* ^Dex20^ = 120 g/L, named “crowded conditions”) and in bacterial cell lysate (*c* ^lysate^ = 120 g/L CAG 18 455 7371, named “cell lysate conditions”). The results obtained here suggest that the intermolecular distance determined for the *Bs*CspB‐dT4 complex does not change significantly when the three different experimental conditions—diluted, crowded, and cell lysate—are compared among each other.

## 2 Results and Discussion

### 2.1 Paramagnetic Spin Labeling of S11C‐*Bs*CspB

Serine at position 11 in the primary sequence of *Bs*CspB was selected as mutation site for the incorporation of a cysteine based on two rationales: (i) the location within the loop between *β*‐sheets 2 and 3 ensures steric availability for the conjugation with the subsequently attached paramagnetic and diamagnetic spin labels and (ii) the distance *d* between Ser11 and the binding site of dT4 falls within the range to be quantified by using ^19^F‐based PRE experiments (14 Å ≤ *d* ≤ 24 Å, according to PDB ID 2ES2 that reports on the complex between *Bs*CspB and dT6). Next, the overall thermodynamic stability Δ*G* ^0^ of S11C‐*Bs*CspB as well as the binding affinity toward dT4 has been determined using fluorescence spectroscopy applying diluted conditions. Quantitatively, the overall thermodynamic stability has been determined to be Δ*G* ^0^ = 10.4 ± 0.5 kJ mol^−1^ while following the folding‐to‐unfolding transition induced by increasing amounts of urea. The comparison to corresponding wild type (wt) data Δ*G* ^0^ = 9.4 ± 0.5 kJ mol^−1^ reveals no significant change (Figure S1). The binding affinity of S11C‐*Bs*CspB toward dT4 did also not change significantly, possessing a dissociation constant of *K*_D_ = 6.7 ± 0.1 µM compared to *K*_D_ = 6.2 ± 0.2 µM determined for the interaction between dT4 and wt *Bs*CspB (Figure S1). Therefore, it can be assumed that the serine‐to‐cysteine mutation at primary sequence position 11 does not alter inherent features of *Bs*CspB concerning the interaction to dT4.

Next, two different paramagnetic spin labels and respective diamagnetic reference compounds were covalently attached to S11C‐*Bs*CspB using the thiol group of the newly introduced cysteine residue: firstly, paramagnetic 3MPrx and diamagnetic maleimide (dMal) and, secondly, 2,2′, 2″‐[10‐[2‐[[2‐(2,5‐dioxo‐2,5‐dihydro‐1*H*‐pyrrol‐1‐yl)ethyl]amino]‐2‐oxoethyl]−1,4,7,10‐tetraazacyclododecane‐1,4,7‐triyl]triacetic acid (maleimido‐mono‐amide‐DOTA) either loaded with paramagnetic Gd(III) (Gd‐DOTA) or diamagnetic Y(III) (Y‐DOTA). In all cases, the labels have been covalently attached to S11C‐*Bs*CspB, permitting a potential cleavage under reductive conditions. The two sets of paramagnetic spin labels were utilized due to known differences regarding stability under cell‐like conditions. While the nitroxide is readily reduced in cell lysate within minutes, Gd(III) remains stable even for years under such conditions [28, 29].

**FIGURE 1:**
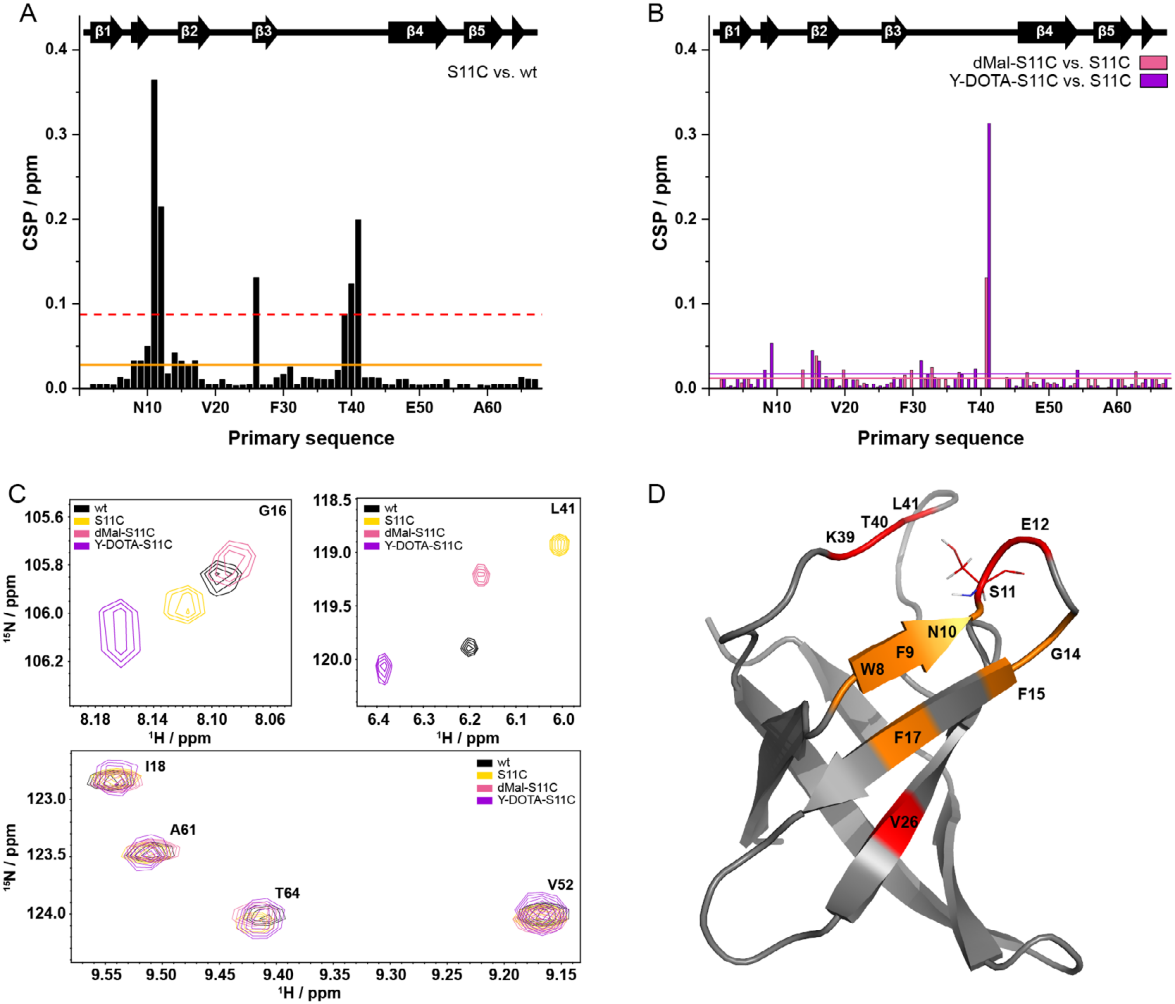
Chemical shift perturbation (CSP) mapping to structurally characterize *Bs*CspB variants. (A) CSPs based on chemical shift values determined for S11C‐*Bs*CspB and compared to wt *Bs*CspB. The continuous line (in orange) represents the mean, and the dashed line (in red) represents the mean plus one standard deviation. Secondary structural elements according to PDB ID 1NMG are indicated at the top. (B) CSPs based on chemical shift values determined for dMal‐S11C‐*Bs*CspB (in salmon) as well as Y‐DOTA‐S11C‐*Bs*CspB (in magenta) and individually compared to S11C‐*Bs*CspB. The horizontal lines represent the mean regarding dMal‐S11C‐*Bs*CspB (in salmon) as well as Y‐DOTA‐S11C‐*Bs*CspB (in magenta). Secondary structural elements according to PDB ID 1NMG reporting on wt *Bs*CspB are indicated at the top. (C) Sections of 2D ^1^H‐^15^N HSQC NMR spectra including backbone assignment of residues corresponding to wt *Bs*CspB (in black), S11C‐*Bs*CspB (in yellow), dMal‐S11C‐*Bs*CspB (in salmon), and Y‐DOTA‐S11C‐*Bs*CspB (in magenta). The assignment presented uses the one letter code for amino acids followed by the position in the primary sequence. The 2D NMR spectra have been acquired at *T* = 298 K and *B*_0_ = 18.8 T. (D) Cartoon representation of wt *Bs*CspB (PDB ID: 1NMG) highlighting residues that exhibit CSPs larger than the mean (in orange), as the mean plus one standard deviation (in red) as determined for the S11C mutation within *Bs*CspB (see panel A).

Two‐dimensional (2D) ^1^H‐^15^N heteronuclear single quantum coherence (HSQC) NMR spectra of wt *Bs*CspB, S11C‐*Bs*CspB, dMal‐S11C‐*Bs*CspB, and Y‐DOTA‐S11C‐*Bs*CspB (Figures S2–S4) have been acquired to evaluate potential structural consequences that may accompany the serine‐to‐cysteine mutation and the subsequent attachment of a spin label. The determination of chemical shift perturbations (CSPs) for S11C‐*Bs*CspB, dMal‐S11C‐*Bs*CspB, and Y‐DOTA‐S11C‐*Bs*CspB regarding chemical shift values observed for wt and S11C‐*Bs*CspB, respectively, enables to quantitatively assess potential structural effects (Figure 1A,B). Individual cross‐peaks of the four different protein species that arise in the 2D ^1^H‐^15^N HSQC NMR spectra are presented in Figure 1C. Most residues that exhibit CSPs of significance (CSP is larger than the mean plus one standard deviation) are found in close proximity to primary sequence position 11. It is also noted that Val26 experiences a CSP of significance, potentially attributed to an allosteric effect that is transmitted via residues Trp8 and Phe17, which also experience modest CSPs (Figure 1D).

Subsequently, the attachment of the paramagnetic as well as the diamagnetic spin label to S11C‐*Bs*CspB was probed by analyzing signal heights of individual cross‐peaks in 2D ^1^H‐^15^N HSQC NMR spectra of 3MPrx‐S11C‐*Bs*CspB and dMal‐S11C‐*Bs*CspB, respectively. For the paramagnetic case, significant attenuation in signal height was observed for cross‐peaks that represent residues spatially close to the spin label (Figure S5A). The same approach was applied for Gd‐DOTA‐S11C‐*Bs*CspB and Y‐DOTA‐S11C‐*Bs*CspB, resulting in a comparable outcome (Figure S5B).

**FIGURE 2:**
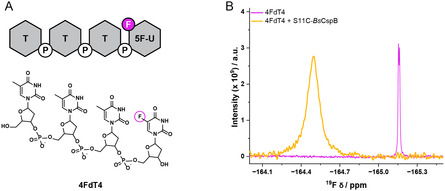
Fluorine‐labeled derivative of dT4, named 4FdT4. (A) Fluorine placed at ring position 5 (in magenta) replaces a methyl group. Thymine and 5‐fluorouracil residues are represented as hexagons (in gray), and the deoxyribose and phosphate backbone are also shown. (B) One‐dimensional ^19^F NMR spectra acquired for 4FdT4 in the absence (in magenta) and presence of a three times stoichiometric excess of S11C‐*Bs*CspB (in orange). The data have been acquired at *T* = 298 K and *B*_0_ = 18.8 T.

Fluorine has been strategically placed into the ligand molecule dT4 used here, finally enabling the acquisition of intermolecular protein‐to‐ligand ^19^F PREs. Specifically, the terminal thymine of dT4 was changed to a 5‐fluorouracil residue (5F‐U), which replaces a methyl group at ring position 5 with a fluorine nucleus, naming the ligand molecule 4FdT4 (Figure 2A). One‐dimensional (1D) ^31^P NMR spectra acquired for dT4 and 4FdT4 reveal no differences of significance in resonance signals evaluating line widths and range of chemical shifts comparing the two molecules (Figure S6A). This suggests that replacing a methyl group of dT4 with a fluorine atom does not perturb inherent structural and dynamic characteristics of this molecule.

The interaction between the polythymidines and *Bs*CspB is primarily facilitated by pi‐stacking and hydrogen bond interactions between the nucleobases and specific residues in *Bs*CspB [30]. Consequently, no significant change in binding affinity between dT4 and *Bs*CspB is anticipated when dT4 is changed to 4FdT4 due to the strategic choice of the site used for fluorine labeling. Clearly, comparing 1D ^19^F NMR spectra acquired for 4FdT4 in the absence and presence of a threefold stoichiometric excess of S11C‐*Bs*CspB applying diluted conditions demonstrates a significant downfield shift of the resonance signal (Δ*ω* ≃ 0.65 ppm) and severe line broadening (*FWHM* ^4FdT4^ = 12.8 Hz compared to *FWHM* ^4FdT4+S11C‐*Bs*CspB^ = 78.9 Hz), indicating reduced overall tumbling of the 4FdT4‐S11C‐*Bs*CspB complex compared to individual 4FdT4 (Figure 2B).

A similar observation was made for the acquisition of 1D ^19^F NMR spectra applying crowded conditions, also indicating a prominent interaction between 4FdT4 and S11C‐*Bs*CspB (Figure S6). The acquisition of ^19^F NMR spectra of 4FdT4 in cell lysate proved to be challenging due to degradation of DNA fragments in cell lysate. However, the presence of S11C‐*Bs*CspB improved the molecular stability of 4FdT4 to a time scale of several hours at room temperature in cell lysate, highlighting the specificity of this interaction (Figure S6B).

### 2.2 ^19^F Transverse Relaxation Rate Constant *R*_2_ and PRE Enhancement Factor Γ_2_

**FIGURE 3:**
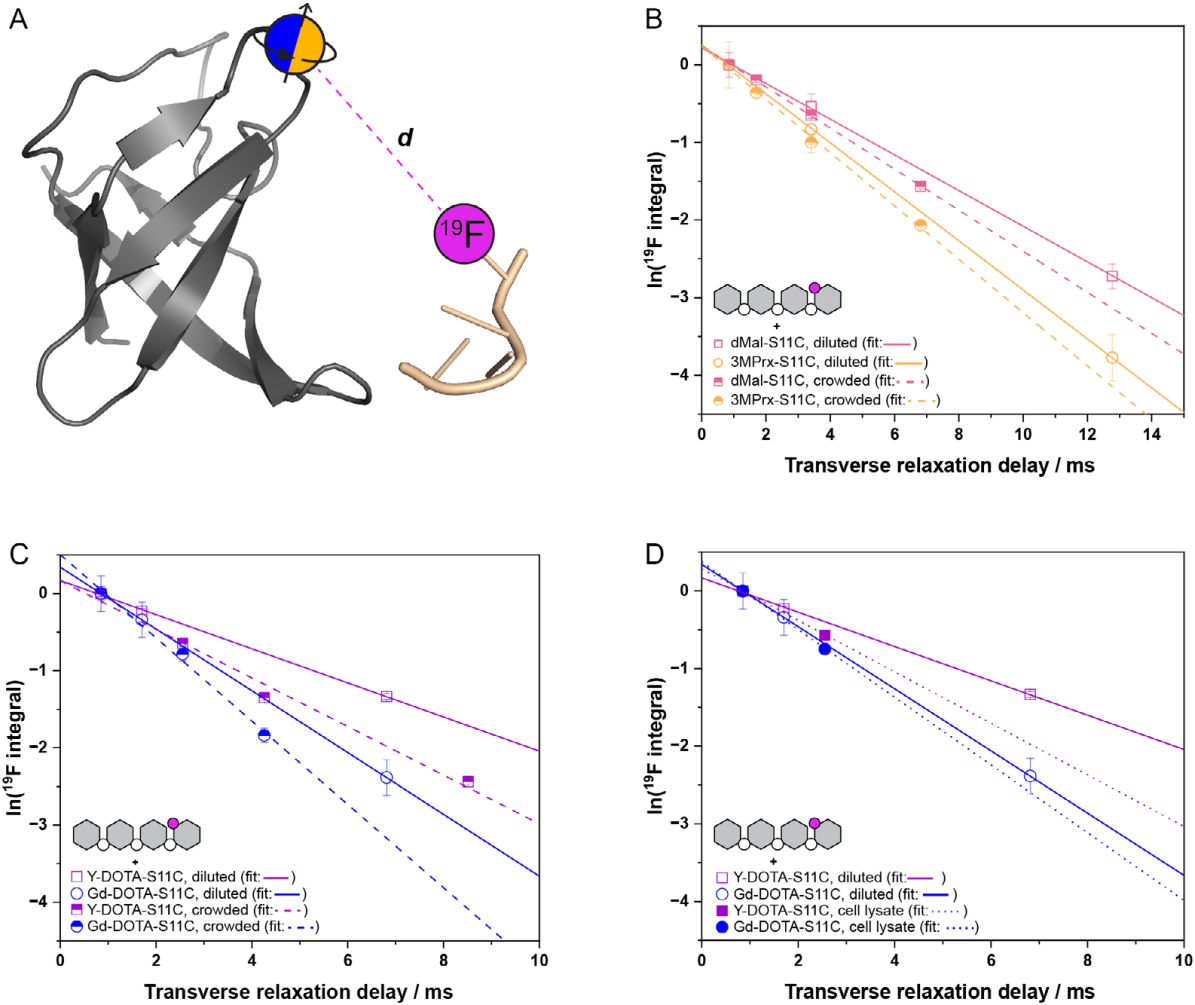
Determination of ^19^F transverse relaxation rate constants and PRE enhancement rates. (A) Design of the molecular complex investigated in this study to determine the distance *d* between spin‐labeled S11C‐*Bs*CspB (cartoon mode, in gray; spin label is highlighted in orange (3MPrx, dMal) and in blue (Gd‐DOTA, Y‐DOTA), and fluorine‐labeled 4FdT4 (light orange). (B) The logarithm of the relative integral of ^19^F comprising 4FdT4 when in complex with 3MPrx‐S11C‐*Bs*CspB (paramagnetic, squares, orange) or dMal‐S11C‐*Bs*CspB (diamagnetic, circles, magenta), determined using increasing lengths of the CPMG relaxation delay. Experiments performed applying diluted (open symbols) or crowded conditions (semi‐filled symbols) are indicated. (C,D) The logarithm of the relative integral of ^19^F comprising 4FdT4 when in complex with Gd‐DOTA‐S11C‐*Bs*CspB (paramagnetic, circles, blue) or Y‐DOTA‐S11C‐*Bs*CspB (diamagnetic, squares, purple) determined using increasing lengths of the CPMG relaxation delay. Experiments performed under diluted (open symbols), crowded (semi‐filled symbols), or in cell lysate conditions (filled symbols) are indicated. The data have been acquired at *T* = 298 K and *B*_0_ = 18.8 T.

In the next step, transverse relaxation rate constants *R*_2_ of 4FdT4 in the presence of the differently spin‐labeled S11C‐*Bs*CspB variants were determined (Figure 3A), while applying the different experimental conditions: diluted, crowded, and cell lysate (Figure 3B–D).

In diluted conditions, 4FdT4 in complex with dMal‐S11C‐*Bs*CspB or Y‐DOTA‐S11C‐*Bs*CspB leads to ^19^F transverse relaxation rate constants that are in good accordance with each other, possessing *R*_2,diluted_ (dMal) = 230 ± 5 s^−1^ and *R*_2,diluted_ (Y‐DOTA) = 220 ± 5 s^−1^ (Table 1). However, in the presence of *c* ^Dex20^ = 120 g/L, the relaxation rate constants increase each (*R*_2,crowded_ (dMal) = 265 ± 5 s^−1^ and *R*_2,crowded_ (Y‐DOTA) = 320 ± 30 s^−1^, Table 1). This might be explained by the increased viscosity of the solvent when switching from diluted to crowded conditions [31]. One should also have in mind that the two spin labels used here, dMal and Y‐DOTA, possess specific impacts due to different molecular sizes. This underlines the importance of using suitable diamagnetic references for each spin label. Please note that the rapid reduction of the nitroxide radical in cell lysate prevents its reliable application under these conditions [32].

**TABLE 1:** Determination of the transverse relaxation rate constants *R*_2_ applying ^19^F CPMG NMR experiments on complexes formed between 4FdT4 and differently spin‐labeled S11C‐*Bs*CspB, acquired under different experimental conditions. The relaxation data have been acquired at *T* = 298 K and *B*_0_ = 18.8 T. The PRE enhancement rate Γ_2_ has been determined using Γ_2_ = *R*_2,paramagnetic_ ‐ *R*_2,diamagnetic_.

Spin label	Condition	*R*_2,diamagnetic_/s^−1^	*R*_2,paramagnetic_/s^−1^	Γ_2_/s^−1^
3MPrx	Diluted	230 ± 5	315 ± 5	85 ± 5
Gd‐DOTA	Diluted	220 ± 5	400 ± 5	180 ± 5
3MPrx	Crowded	265 ± 5	340 ± 10	75 ± 5
Gd‐DOTA	Crowded	320 ± 30	540 ± 50	220 ± 30
Gd‐DOTA	Cell lysate	330^a^	435^a^	110 ± 10

a Transverse relaxation rate constants in cell lysate have been determined using two transverse relaxation delays to minimize experimental time under this condition preventing the determination of an uncertainty.

A detailed analysis of the PRE enhancement rate Γ_2_ enables to follow the spatial distance *d* between 4FdT4 and spin‐labeled S11C‐*Bs*CspB (Figure 3A) when diluted, crowded, and cell lysate conditions are compared among each other. While the numerical value of Γ_2_ does not change significantly between diluted and crowded conditions when 3MPrx was used as spin label, more pronounced differences have been observed when Gd‐DOTA was used as spin label.

However, it must be considered that the PRE‐related correlation time *τ*_c_ reports on the paramagnetic center as well as the fluorine nucleus and has a significant impact on the calculated distance *d* according to the Solomon–Bloembergen equation (Equation (1)):

(1) $$\Gamma_{2}=\frac{1}{15}{\left(\frac{\mu_{0}}{4\pi }\right)}^{2}\gamma^{2}g^{2}\mu_{B}^{2}S\left(S+1\right)\frac{1}{d^{6}}\left\{4\tau_{c}+\frac{3\tau_{c}}{1+\omega^{2}\tau_{c}^{2}}\right\}$$

in which *µ*_0_ is the vacuum permeability, *γ* is the gyromagnetic ratio of the fluorine nucleus, *g* is the electron Landé factor, *µ*_B_ is the electron magnetic moment, *S* is the electron spin quantum number (*S*  =  1/2 for 3MPrx and *S* = 7/2 for Gd‐DOTA), and *ω* is the Larmor frequency of fluorine.

Specifically, the parameter *τ*_c_ depends both on the rotational correlation time *τ*_r_ of the molecule probed and on the effective electron relaxation time *τ*_s_ (Equation (2)):

(2) $$\tau_{c}={\left(\tau_{r}^{-1}+\tau_{s}^{-1}\right)}^{-1}$$

**FIGURE 4:**
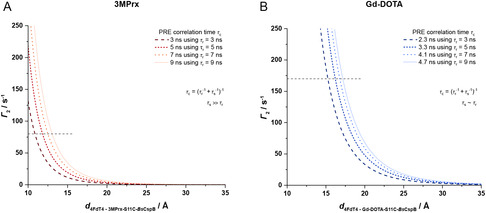
Computed dependence of the PRE enhancement rate Γ_2_ on the distance *d* and rotational correlation time *τ*_r_ using the Solomon–Bloembergen equation. The dashed lines represent the mean value of the experimentally determined PRE enhancement rates Γ_2_ in the present work using the respective spin labels and applying the different experimental conditions. (A) PRE enhancement rate Γ_2_ depends on the distance *d* between the spin label 3MPrx and the affected nucleus, while the rotational correlation time *τ*_r_ of the spin‐labeled molecule varies between 3 and 10 ns. Since the effective electron relaxation time *τ*_s_ is significantly larger than *τ*_r_, the PRE correlation time *τ*_c_ is equal to *τ*_r_. (B) PRE enhancement rate Γ_2_ depends on the distance between the spin label Gd‐DOTA and the affected nucleus, while the rotational correlation time *τ*_r_ of the spin‐labeled molecule varies between 3 and 10 ns. For the calculation of the PRE correlation times *τ*_c_, the effective electron relaxation time *τ*_s_ is assumed to be *τ*_s_ = 10 ns [25].

For Gd‐DOTA, the effective electron relaxation time *τ*_s_ falls within the same range as *τ*_r_ (*τ*_s_ ∼ 10 ns [25]) at a magnetic field strength of *B*_0_ = 18.8 T, as employed in the NMR spectroscopic experiments here, thus possessing a considerable impact on *τ*_c_. In the case of 3MPrx, the numerical value of *τ*_s_ is significantly larger compared to the Gd‐DOTA case. Thus, the approximation *τ*_c_ ≈ *τ*_r_ can be reliably assumed [16]. Also, the viscosity of the solvent impacts the numerical value of *τ*_r_ [33]. As illustrated in Figure 4, changes of the magnitude of the rotational correlation time falling within the range 3 ns ≤ *τ*_r_ ≤ 10 ns exert a considerably more pronounced impact on Γ_2_ for short distances *d* (*d* ≤ 20 Å (3MPrx), *d* ≤ 25 Å (Gd‐DOTA)) between the spin label and the affected probed nucleus than for longer distances. Consequently, the knowledge of the numerical value of *τ*_r_ emerges as a critical factor when PRE NMR spectroscopic data that have been obtained using different experimental conditions are compared among each other.

Consequently, the rotational correlation time *τ*_r_ of the protein–ligand complex was determined experimentally applying diluted as well as crowded conditions. Thus, longitudinal and transverse ^15^N relaxation rate constants *R*_1_ and *R*_2_ of the complex formed between *Bs*CspB and dT4 were determined as outlined in the supporting information (Figure S10). The rotational correlation time *τ*_r_ was derived by numerically solving equation (Eq. S6) for *τ*_r_ [34, 35], resulting in *τ*_r,diluted_ = 4.5 ± 0.1 ns and *τ*_r,crowded_ = 6.9 ± 0.2 ns, respectively (Figure S10). Notably, *τ*_r_ determined for diluted conditions here is in perfect agreement with the reported value for the complex formed between wt *Bs*CspB and heptathymidine (dT7) also applying diluted conditions (*τ*_r_ = 4.4 ns) [30].

Ye and coworkers reported that the cytoplasmic viscosity is about two to three times larger than that of water [36]. Köhn and coworkers showed that the viscosity of water increases by a factor of about four if *c*  =  120 g/L Dex20 is added to the experimentally used buffer solution [31]. Accordingly, the rotational correlation time in cell lysate *τ*_r,lysate_ is assumed to be in between the numerical values determined for diluted (*τ*_r,diluted_ = 4.5 ± 0.1 ns) and crowded conditions (*τ*_r,crowded_ = 6.9 ± 0.2 ns). These two numbers are then used for estimating a range for the distance *d* that is present between 4FdT4 and spin‐labeled S11C‐*Bs*CspB in cell lysate (Table 2).

**TABLE 2:** PRE enhancement rates Γ_2_, correlation times *τ*_c_, and corresponding distances *d* based on ^19^F intermolecular PRE NMR spectroscopic experiments. Distances *d* and corresponding uncertainties Δ*d* are determined using Equation (1).

Spin label	Condition	Γ_2_/s^−1^	*τ*_c_/ns	*d*/Å
3MPrx	Diluted	85 ± 5	4.5 ± 0.1	12 ± 1
3MPrx	Crowded	75 ± 5	6.9 ± 0.2	13 ± 1
Gd‐DOTA	Diluted	180 ± 5	3.1 ± 0.1	16 ± 1
Gd‐DOTA	Crowded	220 ± 30	4.1 ± 0.1	16 ± 2
Gd‐DOTA	Cell lysate	110 ± 10	3.1 ± 0.1 … 4.1 ± 0.1	17 ± 2 … 18 ± 2

### 2.3 Determination of Ligand‐to‐Protein Distances

The intermolecular distance *d* between spin‐labeled S11C‐*Bs*CspB and fluorine containing 4FdT4 was finally calculated according to the Solomon–Bloembergen equation (Equation (1)) based on numerical values for the PRE enhancement rate Γ_2_ and the rotational correlation time *τ*_r_ (see above). The results obtained for *d* while applying different experimental conditions are summarized in Table 2.

Evaluation of the calculated distances reveals that the intermolecular distances determined for the complex formed between Gd‐DOTA‐S11C‐*Bs*CspB and 4FdT4 are larger than those obtained for the complex formed between 3MPrx‐S11C‐*Bs*CspB and 4FdT4. This data implies that the two spin labels 3MPrx and Gd‐DOTA differ in orientation regarding 4FdT4. While the spin label 3MPrx is rather small (*M*_W_ = 239.3 g/mol) possessing rather limited rotational freedom, the spin label Gd‐DOTA is considerably bulkier (*M*_W_ = 681.8 g/mol) and possesses larger rotational freedom. In addition, the substantially longer tether of Gd‐DOTA is more hydrophilic than the tether of 3MPrx, which may favor specific conformers which then lead to different distances. Consequently, differences in calculated distances can be anticipated when Gd‐DOTA‐ and 3MPrx‐labeled S11C‐*Bs*CspB variants are compared among each other.

Comparing the calculated distances between spin‐labeled S11C‐*Bs*CspB and 4FdT4 determined either in diluted or crowded conditions reveals overall comparability. When 3MPrx‐S11C‐*Bs*CspB was employed, the difference in distances to 4FdT4 is about 1 Å with an uncertainty that is inherent to the here chosen experimental setup being in the same range (Table 2). When Gd‐DOTA‐S11C‐*Bs*CspB was employed, the mean distances to 4FdT4 do not significantly differ when diluted and crowded conditions are compared while considering an uncertainty of about 1–2 Å (Table 2). Note that flexibilities of the tethers of the two paramagnetic tags as well as the flexibility of the solvent‐exposed loop comprising S11C‐*Bs*CspB to which they are attached have to be determined individually and precisely if absolute distances—and not differences in distances as here—are specifically in focus.

**FIGURE 5:**
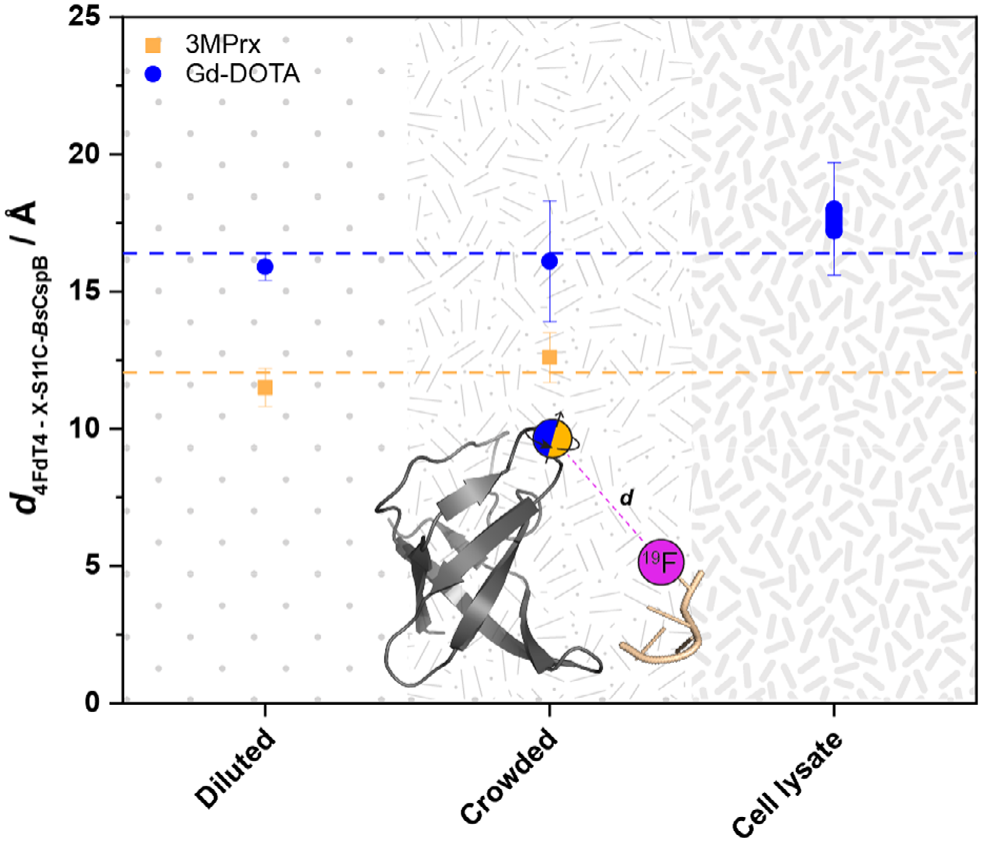
Calculated distances *d* between differently spin‐labeled S11C‐*Bs*CspB and 4FdT4 as determined by ^19^F‐based PREs applying diluted, crowded, and cell lysate conditions. Distances that have been determined by using 3MPrx as spin label are shown as squares (in orange), whereas distances that have been determined by using Gd‐DOTA as spin label are shown as circles (in blue). The letter X codes for the spin label applied. The horizontal lines (dashed mode) represent the mean distance *d* observed for using 3MPrx (in orange) or Gd‐DOTA (in blue) as spin labels.

In cell lysate, the calculated distance between Gd‐DOTA‐S11C‐*Bs*CspB and 4FdT4 appears to be in between *r*_lysate_ = 17 ± 2 Å and *r*_lysate_ = 18 ± 2 Å, depending on the value of *τ*_c_ (see discussion above) used for the calculation (Table 2). Consequently, the distance for the protein–ligand complex probed here when transitioning from diluted to cell lysate conditions is almost conserved, especially when experimental uncertainty is fully considered (Figure 5).

## 3 Conclusion

The precise application of ^19^F PRE NMR spectroscopic methodology facilitates the quantitative determination of intermolecular distances considering different experimental conditions with a manageable time commitment. Following this strategy, quantitative distance information can be reliably determined in diluted, crowded, and cell lysate conditions. The analysis of the ^19^F PRE NMR spectroscopic data acquired in the present study indicates that the intermolecular protein‐to‐ligand distance is conserved when the results for applying the three different experimental conditions are compared among each other. We anticipate that the here presented experimental approach will be of high attention for researchers interested in structural characteristics of biomolecular complexes that are present at rather intricate conditions.

## Supporting Information

Additional supporting information can be found online in the Supporting Information section. The authors have cited additional references within the Supporting Information [30, 31, 34, 35, 37–40].

## Funding

This work was supported by Deutsche Forschungsgemeinschaft (KO4687/4‐1).

## Conflicts of Interest

The authors declare no conflicts of interest.

## Supporting information

Supplementary Material
