# Supplementary material for: Quantifying Ligand‐to‐Protein Distances in Complex Environments Using Intermolecular 19F PRE NMR Spectroscopy
Source: Chembiochem. 2026 Apr 17;27(8):e70309. doi: 10.1002/cbic.70309 (PMC13089942; doi:10.1002/cbic.70309)
Supplement: Supplementary file 1 — Supplementary Material [file CBIC-27-e70309-s001.pdf]

Supporting Information

Quantifying ligand-to-protein distances in complex environments using intermolecular ^{19}F PRE NMR spectroscopy

Yannick Werle[a,b], Martha-Louise Inderfurth[a], Christopher J. Lang[a], Michael Kovermann[a,b]*

[a] Y. Werle, M. Inderfurth, C. J. Lang, M. Kovermann
Department of Chemistry
Universität Konstanz
Universitätsstraße 10, 78464 Konstanz, Germany

[b] Y. Werle, M. Kovermann
Graduate School of Chemical-Biology (KoRS-CB)
Universität Konstanz
Universitätsstraße 10, 78464 Konstanz, Germany

*E-mail: michael.kovermann@uni-konstanz.de

Table of Contents

Experimental Section 3

Protein Expression and Purification 3

Bioorthogonal labeling reactions 3

Fluorescence spectroscopy 3

Preparation of cell lysate 4

Sample preparation 4

NMR spectroscopy 5

^{19}F R_2 measurement and PRE experiments 5

Determination of the rotational correlation time 6

Supporting Figures 7

References 17

Experimental Section

Protein Expression and Purification

The vector pET11a carrying the primary sequence of *Bacillus subtilis* cold shock protein B (*Bs*CspB) with serine at position 11 being exchanged to cysteine was gratefully obtained from L. Williams and M. Adams (University of Konstanz, Drescher group). Protein expression and purification have been performed as described previously except for the addition of dithiothreitol (DTT) to a final concentration of c^{DTT} = 1 mM to all buffers used for protein purification.[1-4] Protein concentration has been determined by UV/Vis spectroscopy using an extinction coefficient of S11C-*Bs*CspB of $\varepsilon^{280\ nm}$ = 5800 M^{-1} cm^{-1}. Isotopic ^{15}N-labeling was achieved by performing the expression in M9 minimal medium using ^{15}N-ammonium chloride (obtained from Cortecnet) as the nitrogen source.

Bioorthogonal labeling reactions

For protein labeling with 3-maleimido-PROXYL (3MPrx, obtained from Merck) and maleimide (dMal, obtained from Merck), 100 µM purified S11C-*Bs*CspB in phosphate buffer (20 mM Na_2HPO_4 pH = 7.0) were incubated with a 5-fold stochiometric excess of the spin label, added from a 100 mM stock solution in DMSO, for 16 h with vigorous shaking at 8 °C. The tagged protein was purified using centrifugal filters with a 3 kDa cut off ('Amicons', obtained from Merck) and subsequently the concentration was determined by UV/Vis spectroscopy using an extinction coefficient of $\varepsilon^{280\ nm}$ = 5800 M^{-1} cm^{-1}.

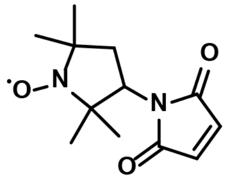

3MPrx

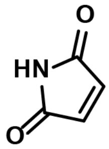

dMal

For protein labeling with maleimido-mono-amide-DOTA (DOTA-M, obtained from TCI Germany), firstly DOTA-M was incubated with 1.1 equivalents of $GdCl_3$ (obtained from Merck) or YCl_3 (obtained from Merck) applying vigorous shaking at 37 °C for 4 h. Subsequently, 0.2 equivalents of purified S11C-*Bs*CspB in phosphate buffer (20 mM Na_2HPO_4 pH = 7.0) were added and the mixture was incubated for 16 h applying vigorous shaking at 8 °C. Purification and determination of concentration were performed as described above.

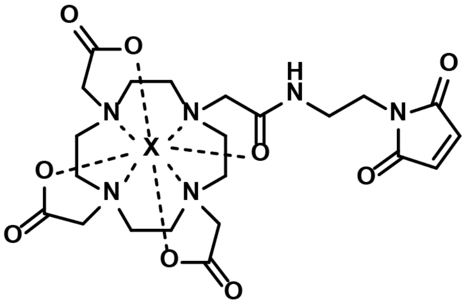

X-DOTA-M

Fluorescence spectroscopy

Fluorescence spectroscopy experiments in equilibrium were performed to probe overall thermodynamic stability and binding of dT4 to S11C-*Bs*CspB by a FP-8500 spectrofluorometer (Jasco).

For urea induced folding-to-unfolding experiments, the final protein concentration was set to c = 1 µM in phosphate buffer (20 mM Na_2HPO_4 pH = 7.0). The folding-to-unfolding transitions were monitored between 0 M ≤ c_{urea} ≤ 8 M by acquiring at least 32 data points. After mixing, samples were thermally equilibrated for at least 30 minutes and measured under stirring conditions at a temperature of T = 298 K, maintained by a water bath. Fluorescence spectra were acquired as technical triplicates upon excitation at λ = 280 nm in the range from 290 nm to 400 nm in 0.5 nm steps. After each acquired data point, the refractive index of the sample was measured to determine the urea concentration according to following equation:

$$c_{\mathrm{urea}} = 117.66 \cdot \Delta n + 29.753 \cdot \Delta n^2 + 185.56 \cdot \Delta n^3, \qquad (S1)$$

with Δn representing the difference of the refractive index using samples with and without urea. Furthermore, all fluorescence spectra were background corrected by acquiring spectra at different urea concentrations in absence of protein. For analysis, only intensities between λ = 320 nm and λ = 380 nm were used to determine intensity averaged wavelength (<λ>) of each data point according to equation S2:

$$< \lambda > = \frac{\sum_{\mathrm{i}=1}^{\mathrm{N}}(I_{\mathrm{i}}\lambda_{\mathrm{i}})}{\sum_{\mathrm{i}=1}^{\mathrm{N}}(I_{\mathrm{i}})}, \qquad (S2)$$

with I_{i} being the intensity at wavelength λ_{i}.
The parameter <λ> was then plotted against the urea concentration and fitting of equation S3 to the data has been performed:

$$< \lambda > = \frac{(g_{\mathrm{N}} + m_{\mathrm{N}} \cdot c_{\mathrm{urea}}) + (g_{\mathrm{U}} + m_{\mathrm{U}} \cdot c_{\mathrm{urea}}) \cdot \left(e^{-\frac{\Delta G^0}{RT}} + \frac{m \cdot c_{\mathrm{urea}}}{RT}\right)}{1 + \left(e^{-\frac{\Delta G^0}{RT}} + \frac{m \cdot c_{\mathrm{urea}}}{RT}\right)}, \qquad (S3)$$

with $g_{\mathrm{N/U}}$ and $m_{\mathrm{N/U}}$ being the slopes and the intersections of the baselines of the folded state and the ensemble of unfolded conformations, respectively, ΔG^0 representing the overall thermodynamic stability at a concentration of c_{urea} = 0 M, R the universal gas constant, T the temperature and m the cooperativity of unfolding.

For the determination of the binding affinity, titration series with protein concentrations of c = 3.5 µM in phosphate buffer (20 mM Na_2HPO_4 pH = 7.0) were acquired. Oligonucleotide dT4 (obtained from biomers.net) was subsequently added from a 1 mM stock solution in phosphate buffer in 32 titration steps reaching a 30-fold stoichiometric excess of dT4 regarding the protein concentration. Spectra were acquired upon excitation at λ = 280 nm in the range from 290 nm to 400 nm in 0.5 nm steps and as technical triplicates. Fluorescence intensity at λ = 342 nm was read out and equation S4 was applied to determine the dissociation constant K_{D} quantifying the binding process:

$$Q = Q_{\max} \cdot \frac{A - \sqrt{A^2 - 4n \cdot [P]_0 \cdot [Y]_0}}{2 \cdot [P]_0}, \qquad (S4)$$

with $A = K_{\mathrm{D}} + [P]_0 + n[Y]_0$, Q is the quenching of the intrinsic fluorescence emission originating from the protein and $[P]_0$ and $[Y]_0$ are the total concentrations of protein and dT4, respectively. The stoichiometry of binding n was set to n = 1 according to literature.[5]

Preparation of cell lysate

For cell lysate preparation, CAG 18455 7371 bacteria in LB medium were harvested at an optical density of $OD_{600} \geq 1.7$ by centrifugation for 30 minutes with 4000 g at T = 4 °C. The cell pellet was weighed and resuspended in V = 1 – 2 mL phosphate buffer (20 mM Na_2HPO_4 pH = 7.0). The volume of buffer added depends on the target concentration of the cell lysate stock solution. The suspension was then disrupted by sonification for 100 s per 1 mL of suspension and subsequently centrifuged for 30 minutes with 4000 g at 4 °C. The supernatant was used to set the CAG cell lysate concentration in the respective samples to $c^{\mathrm{cell\ lysate}}$ = 120 g/L.

Sample preparation

All samples were either measured in pure 20 mM Na_2HPO_4 buffer at pH = 7.0 or in buffer containing 120 g/L Dex20 (obtained from Pharmacosmos) or 120 g/L CAG cell lysate. All samples contained 95 % (*v*/*v*) H_2O and 5 % (*v*/*v*) D_2O. Protein concentration was kept at c^{protein} = 150 µM and 4FdT4 (obtained from biomers.net) concentration at c^{4FdT4} = 50 µM to ensure that all 4FdT4 molecules are bound to the protein. Potential contributions of paramagnetic species in solution to the transverse relaxation rates have been shown to be negligible in the concentration range used in the experiments (Figure S7). For referencing purposes (direct referencing in ^{1}H as indirect referencing in ^{15}N, ^{19}F and ^{31}P dimensions, respectively), TMSP was added to the NMR spectroscopically used samples possessing a concentration of c^{TMSP} = 100 µM. Furthermore, to prevent biodegradation of the samples, sodium azide (NaN_3) was added to the samples to a concentration of c^{azide} = 5 mM. For preparation of samples in presence of molecular crowders, Dex20 was added using a 380 g/L stock solution to a final sample concentration of c^{Dex20} = 120 g/L. Cell lysate samples were prepared by adding CAG lysate from a stock solution prepared as described above to a final concentration of c^{lysate} = 120 g/L.

NMR spectroscopy

Unless stated otherwise, all NMR spectroscopic experiments were carried out on an 800 MHz Bruker NEO NMR spectrometer equipped with a CP-QCI cryogenically cooled probe at T = 298 K sample temperature. One-dimensional ^{19}F NMR spectra were collected with 1368 data points, a spectral width of 10 ppm and a recycle delay of 1.5 s. The carrier frequency was set to −162.8 ppm and the number of scans varied from 8192 to 12288, depending on the signal-to-noise ratio. NMR spectra were processed using TopSpin 4.1.4 (Bruker). Two-dimensional ^{1}H-^{15}N HSQC spectra were collected with 1024 data points in the direct dimension and 256 data points in the indirect dimension using a recycle delay of 1 s. The number of scans varied from 8 to 16, depending on sample concentration. Water suppression occurred using both presaturation and a Watergate pulse sequence. Two-dimensional NMR spectra were processed using NMR pipe[6] and analyzed using NMR view[7]. Perturbations of the chemical shift, $\Delta\omega$, while analyzing ^{1}H-^{15}N HSQC spectra were calculated according to equation S5:

$$\Delta\omega = \sqrt{\frac{(\Delta\delta^{1}H)^2 + \frac{1}{25}(\Delta\delta^{15}N)^2}{2}}, \qquad (S5)$$

with $\Delta\delta^{1}$H representing changes of the ^{1}H chemical shift and $\Delta\delta^{15}$N representing changes of the ^{15}N chemical shift.

For the assignment of the resonance signals in the ^{1}H-^{15}N HSQC spectra of S11C-*Bs*CspB, dMal-S11C-*Bs*CspB and Y-DOTA-S11C-*Bs*CspB, three-dimensional ^{15}N-NOESY-HSQC spectra were acquired. For S11C-*Bs*CspB, the ^{15}N-NOESY-HSQC spectrum was acquired on an Avance III 600 MHz Bruker spectrometer equipped with a triple resonance TCI cryoprobe at T = 298 K sample temperature.
All three-dimensional spectra were collected as a series of 320 data sets in t_2 composed of 88 values in t_3 and 1024 data points with the number of scans varying between 2 and 8, depending on sample concentration. A recycle delay of 1 s was used and the NOE mixing time was set to 100 ms. Water suppression occurred using a Watergate pulse sequence. Three-dimensional NMR spectra were processed and analyzed analog to the two-dimensional NMR spectra.

^{19}F R_2 measurement and PRE experiments

^{19}F R_2 rate constants were determined by CPMG experiments applying four different relaxation delays and two duplicates to determine the standard deviation for diluted and crowded conditions. The delay between successive π-pulses applied in CPMG experiments was set to 400 µs. In CAG cell lysate, CPMG experiments were performed with two different relaxation delays to reduce experimental time.[8] These two relaxation experiments were repeated in reverse order regarding the relaxation delay to estimate the standard deviation. Transverse relaxation delays varied between 0.85 ms and 34.06 ms. One-dimensional ^{19}F NMR spectra and two-dimensional ^{1}H-^{15}N HSQC spectra were acquired before and after the relaxation experiments to ensure sample stability. Relative integrals were determined and the natural logarithm of integrals was calculated and plotted against the transverse relaxation delay. Fitting a line function to the experimental data yielded the numerical value of R_2 and corresponding standard deviation.

The ^{19}F PRE enhancement rates Γ_2 were determined as the difference between the relaxation rate constants obtained for the paramagnetic state ($R_{2,\text{paramagnetic}}$) and for the diamagnetic state ($R_{2,\text{diamagnetic}}$) of the sample, respectively. The intermolecular distance between the ^{19}F nucleus and the spin label was then calculated according to the Solomon-Bloembergen equation (Eq. 1).

Determination of the rotational correlation time

To determine the rotational correlation time τ_r of wt *Bs*CspB when in complex with dT4, ^{15}N longitudinal R_1 and transverse R_2 rate constants were determined by acquiring pseudo 2D ^{1}H-^{15}N HSQC experiments (comprising one increment each). Relative integrals were plotted against the relaxation delay applied: In T_1 relaxation experiments, integrals were obtained in the range between 10 ppm and 6 ppm. To exclude potential contributions from sidechain NH_x groups, in the T_2 relaxation experiments integrals were obtained in the range between 10 ppm and 8 ppm. Subsequently, fitting of a single exponential decay function to the experimental data has been applied. The respective relaxation rate constants have been determined to: $R_{1,\mathrm{diluted}}$ = 1.60 ± 0.03 s^{-1} as $R_{2,\mathrm{diluted}}$ = 7.4 ± 0.3 s^{-1}, and $R_{1,\mathrm{crowded}}$ = 1.20 ± 0.02 s^{-1} as $R_{2,\mathrm{crowded}}$ = 11.0 ± 0.4 s^{-1}, respectively.
These values where than used to derive the rotational correlation time τ_r by numerically solving the following equation[9, 10]:

$$\frac{R_2}{R_1} = \frac{4J(0) + J(\omega_\mathrm{N} - \omega_\mathrm{H}) + 3J(\omega_\mathrm{N}) + 6J(\omega_\mathrm{H}) + 6J(\omega_\mathrm{N} + \omega_\mathrm{H}) + \left(\frac{c^2}{3d^2}\right)\{4J(0) + 3J(\omega_\mathrm{N})\}}{2J(\omega_\mathrm{N} - \omega_\mathrm{H}) + 6J(\omega_\mathrm{N}) + 12J(\omega_\mathrm{N} + \omega_\mathrm{H}) + 2\left(\frac{c^2}{3d^2}\right)J(\omega_\mathrm{N})}, \qquad (S6)$$

with:

$$J(\omega_X) = \frac{2}{5}S^2\left[\frac{\tau_\mathrm{r}}{1 + (\omega_\mathrm{X}\tau_\mathrm{r})^2}\right] \qquad (S7)$$

and:

$$c = \frac{\omega_\mathrm{N}\,\Delta\sigma}{\sqrt{3}} \qquad (S8)$$

and:

$$d = \frac{\mu_0 h \gamma_\mathrm{N}\gamma_\mathrm{H}}{\langle r_\mathrm{NH}^3\rangle(8\pi^2)}, \qquad (S9)$$

in which J is the spectral density function, ω_X is the Larmor frequency of spin X, S^2 is the order parameter (here: S^2 = 1), $\Delta\sigma$ is the chemical shift anisotropy of the N spin ($\Delta\sigma$ = −160 ppm), μ_0 is the vacuum permeability, h is the Planck constant, γ_X is the gyromagnetic ratio of spin X, and r_{NH} is the N-H bond length (r_{NH} = 1.02 Å).

Determination of (macro)viscosities

(Macro)viscosities η have been determined using a rheometer (Hydramotion Viscolite 700) at T = 293 K.

**Condition**	**(Macro)viscosity / cP**
Dilute	0.8 ± 0.1
Crowded (c^{Dex20} = 120 g/L)	3.8 ± 0.1
Cell lysate ($c^{\mathrm{cell\ lysate}}$ = 120 g/L)	1.5 ± 0.1

Supporting Figures

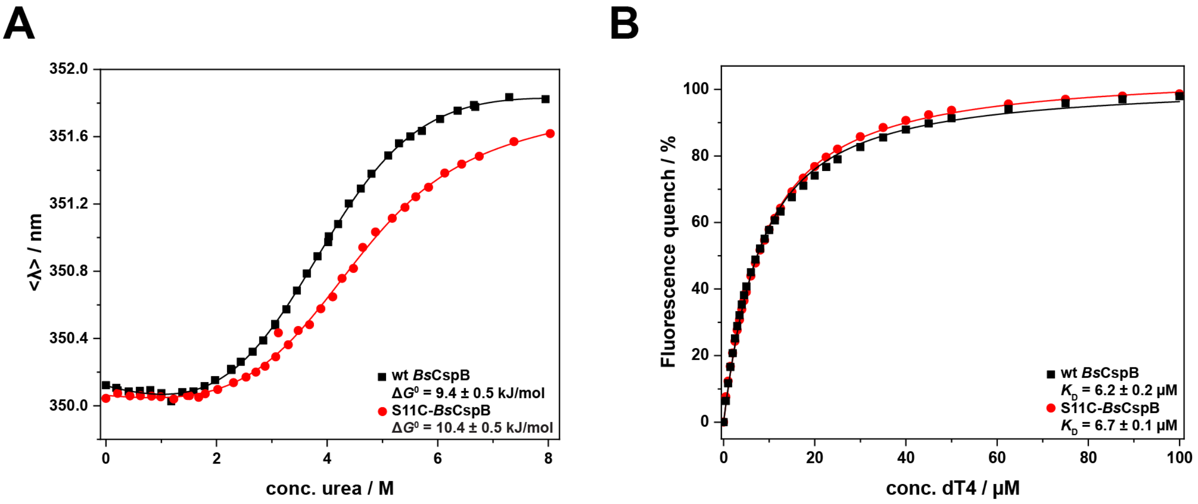

Figure S1. Fluorescence spectroscopy to probe overall thermodynamic stability of S11C-*Bs*CspB and binding affinity of S11C-*Bs*CspB to dT4 compared to wild type *Bs*CspB. A) Intensity averaged wavelength <λ> plotted against the concentration of urea to determine the overall thermodynamic stability ΔG^0. The parameter <λ> was employed to compensate for the shift of the maximum of intrinsic fluorescence emitted by tryptophan due to increasing urea concentrations.[11] There is no significant difference in the ΔG^0 value when wt *Bs*CspB and S11C-*Bs*CspB are compared among each other. B) Quenching of fluorescence emission originating from the protein sample upon addition of dT4 to determine the dissociation constant K_D reporting on binding. No significant difference in the K_D value between wt *Bs*CspB and S11C-*Bs*CspB has been observed.

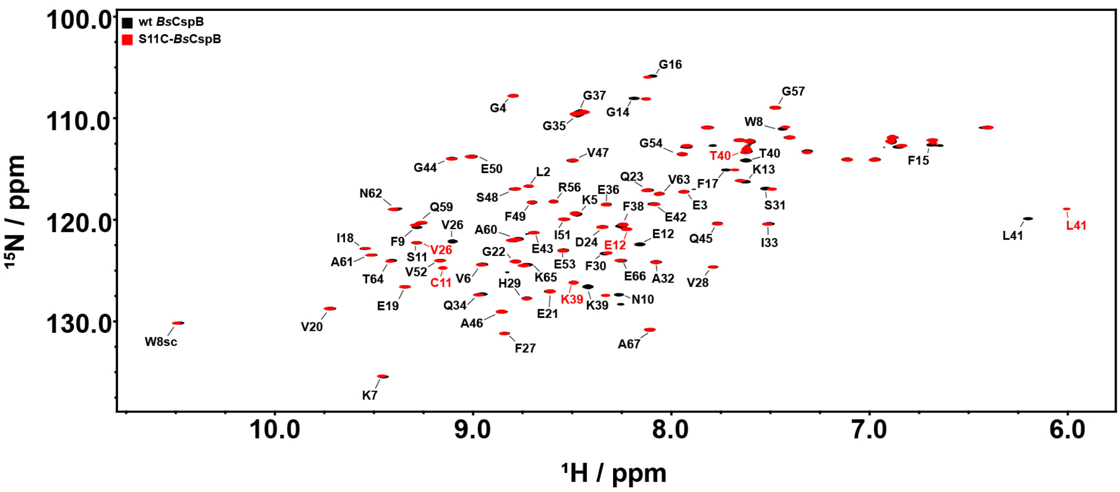

Figure S2. Two-dimensional ^{1}H-^{15}N HSQC spectra of wt *Bs*CspB (black) and S11C-*Bs*CspB (red). Assignment of wt *Bs*CspB is indicated by one letter code used for amino acids followed by the position in the primary sequence. Assignment of residues comprising S11C-*Bs*CspB that differs significantly in chemical shift values to wt *Bs*CspB is indicated using red color for the labelling. The data have been acquired at T = 298 K using B_0 = 18.8 T.

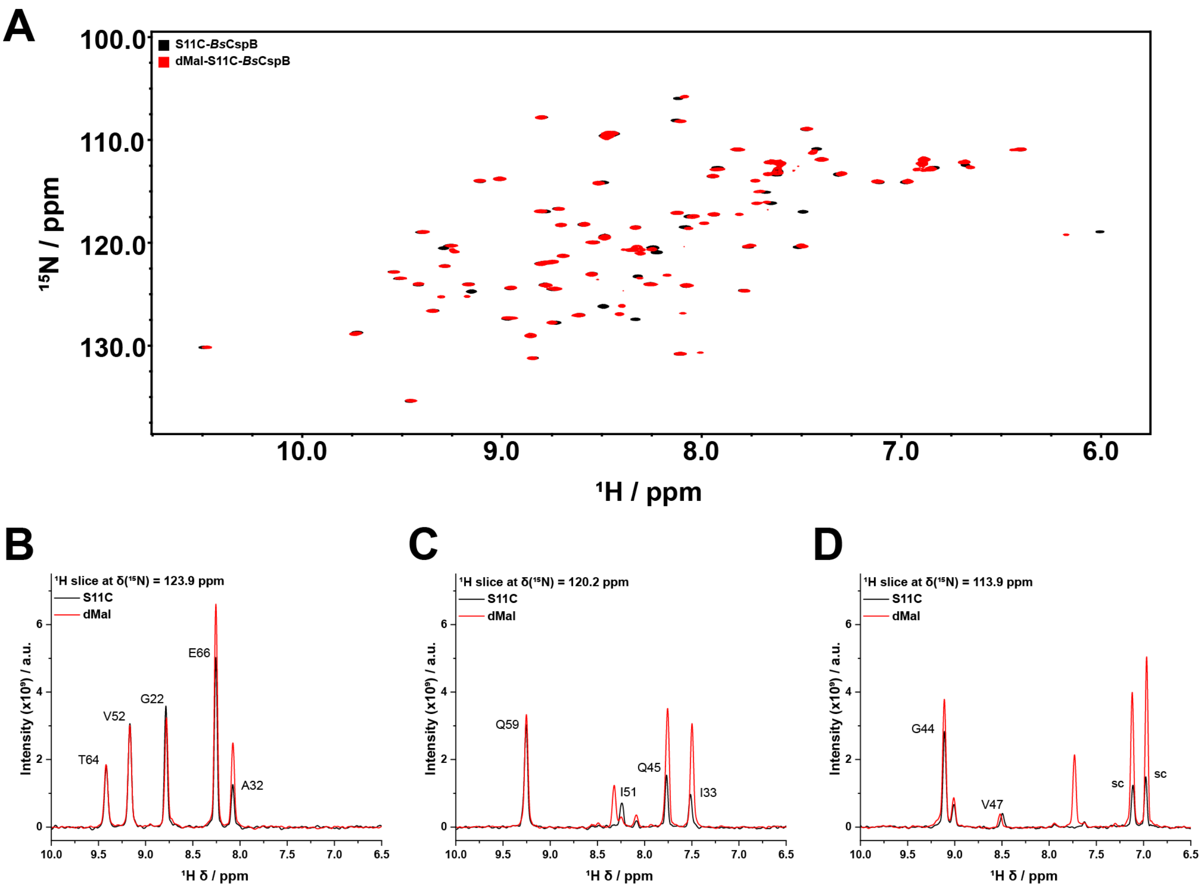

Figure S3. A) 2D ^{1}H-^{15}N HSQC NMR spectra recorded for S11C-*Bs*CspB (black) and dMal-S11C-*Bs*CspB (red). The data have been acquired at T = 298 K using B_0 = 18.8 T. B) - D) One-dimensional ^{1}H spectra obtained from ^{1}H-^{15}N HSQC spectra shown in A) using specific ^{15}N chemical shifts δ as indicated. Resonance signals are assigned using the one letter code of amino acids and the position in the primary sequence. Resonance signals originating from side chain nuclei are abbreviated using "sc".

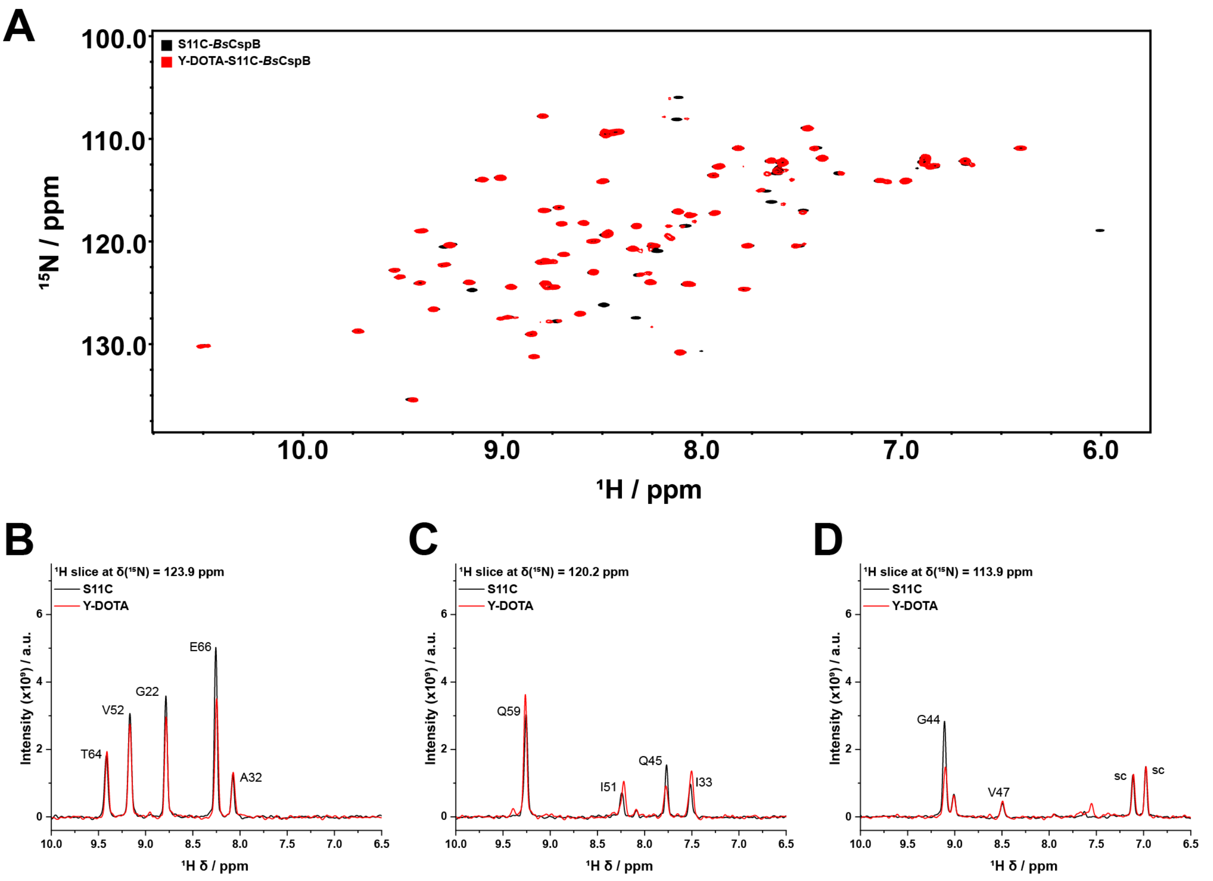

Figure S4. 2D ^{1}H-^{15}N HSQC NMR spectra recorded for S11C-*Bs*CspB (black) and Y-DOTA-S11C-*Bs*CspB (red). The data have been acquired at T = 298 K using B_0 = 18.8 T B) - D) One-dimensional ^{1}H spectra obtained from ^{1}H-^{15}N HSQC spectra shown in A) using specific ^{15}N chemical shifts δ as indicated. Resonance signals are assigned using the one letter code of amino acids and the position in the primary sequence. Resonance signals originating from side chain nuclei are abbreviated using "sc".

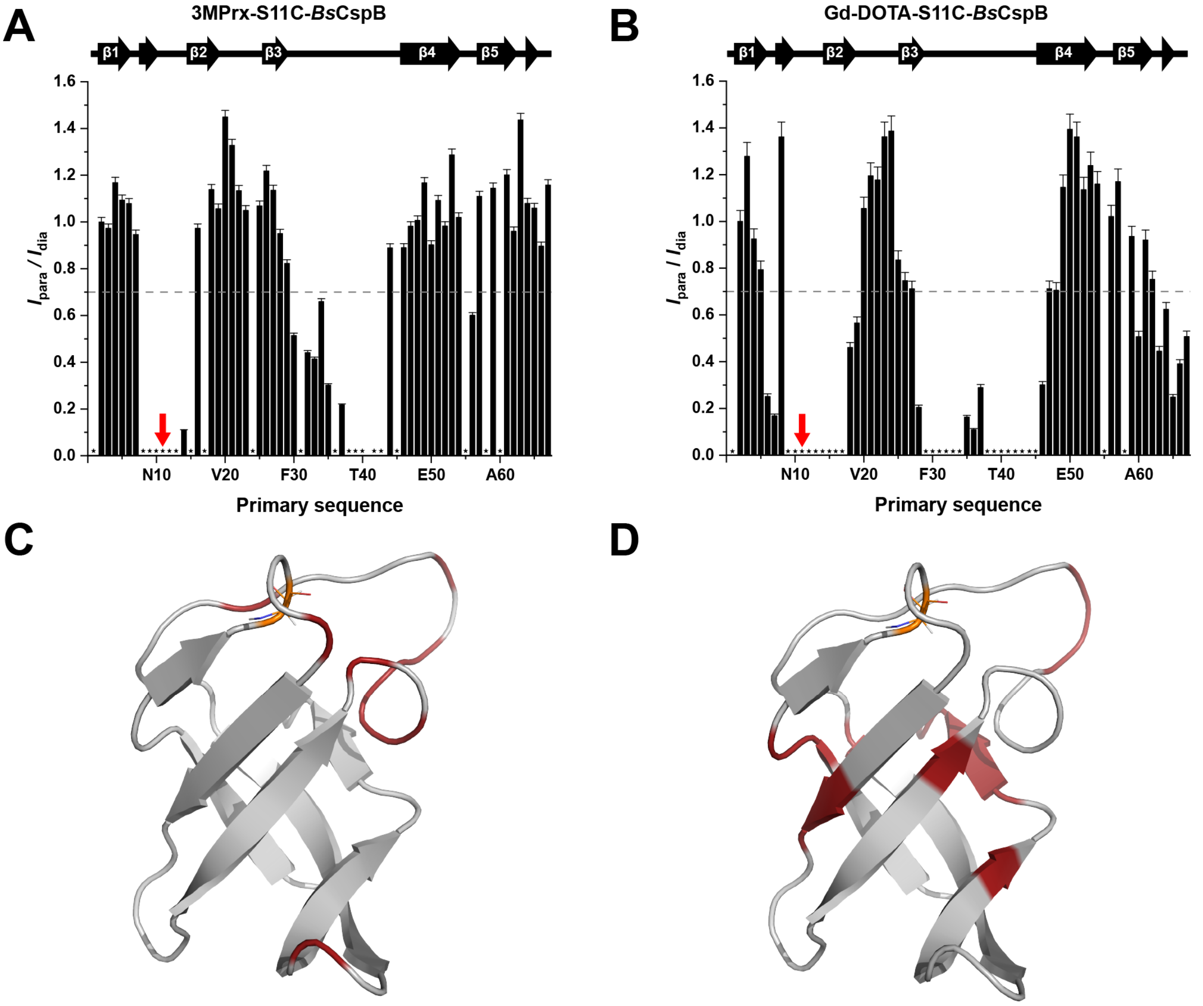

Figure S5. Ratio of signal heights reporting on $^{1}H_{N}$-PREs. At the top, the secondary structural elements of *Bs*CspB (using PDB ID: 1NMG) are indicated. Positions in the primary sequence that lack experimental data (mainly due to ambiguous assignment of backbone resonances) are indicated by asterisks. Position 11 of the primary sequence comprising S11C-*Bs*CspB is highlighted by an arrow colored in red. The signal-to-noise ratio was used to determine error bars, using a signal-to-noise ratio of 100 corresponding to a relative error of 1 %. A) Ratio determined for 3MPrx-S11C-*Bs*CspB (paramagnetic case) and dMal-S11C-*Bs*CspB (diamagnetic case). B) Ratio determined for Gd-DOTA-S11C-*Bs*CspB (paramagnetic case) and Y-DOTA-S11C-*Bs*CspB (diamagnetic case). C) Cartoon representation of wild type *Bs*CspB (PDB ID 1NMG) highlighting position S11 (line mode, in orange color) and residues possessing a ratio of signal heights $0 < I_{para}/I_{dia} \leq 0.7$ determined for 3MPrx-S11C-*Bs*CspB (in red color). D) Cartoon representation of wild type *Bs*CspB (PDB ID 1NMG) highlighting position S11 (line mode, in orange color) and residues possessing a ratio of signal heights $0 < I_{para}/I_{dia} \leq 0.7$ determined for Gd-DOTA-S11C-*Bs*CspB (in red color).

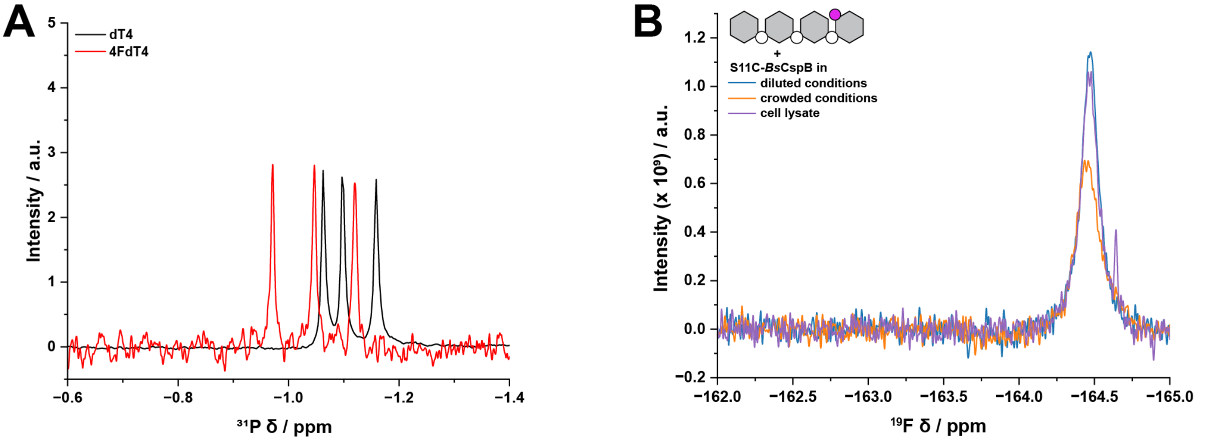

Figure S6. A) One-dimensional ^{31}P NMR spectra recorded for dT4 (colored in black) and 4FdT4 (colored in red) at B_0 = 18.8 T and T = 298 K while applying dilute conditions. The concentration of dT4 was set to c^{dT4} = 1 mM and the concentration of 4FdT4 was set to c^{4FdT4} = 50 µM while 128 as 1024 scans, respectively, have been used for data acquisition. B) One-dimensional ^{19}F NMR spectra recorded for 4FdT4 in presence of S11C-*Bs*CspB applying different experimental conditions: diluted (colored in blue), crowded (colored in orange) and cell lysate (colored in violet). In cell lysate, a sharp resonance signal ($\delta \simeq$ -164.6 ppm) is observed, which is likely to originate from degradation of 4FdT4 under these conditions. The data have been acquired at T = 298 K using B_0 = 18.8 T. The three spectra have been acquired with the same concentrations of 4FdT4 of c^{4FdT4} = 50 µM and S11C-*Bs*CspB of $c^{\mathrm{S11C\text{-}}Bs\mathrm{CspB}}$ = 150 µM and using 2048 scans.

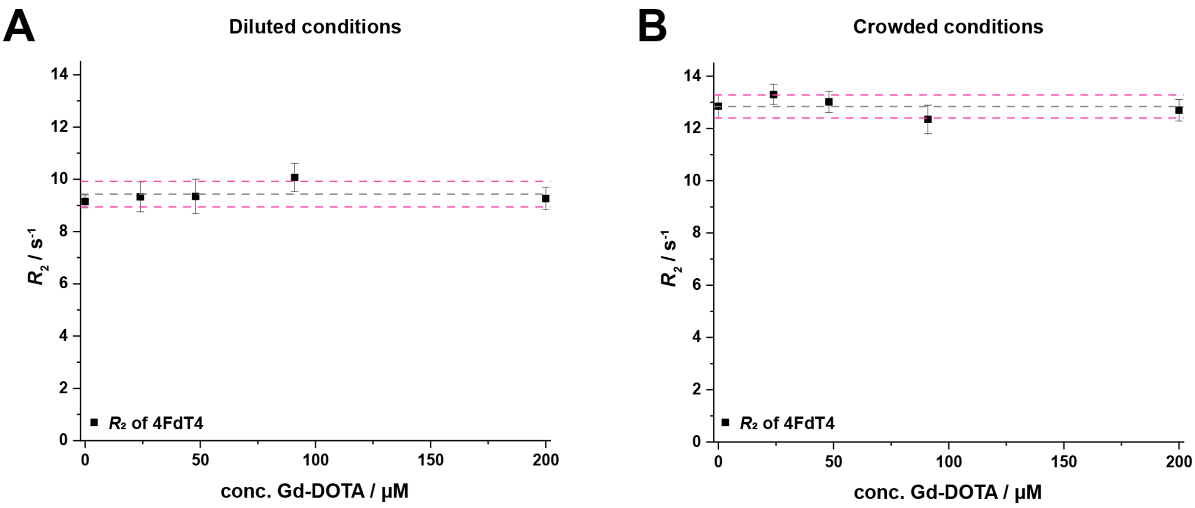

Figure 7. ^{19}F transverse relaxation rate constants R_2 determined for 4FdT4 acquired at different concentrations of the paramagnetic spin label Gd-DOTA, applying different experimental conditions. The dashed lines (in grey color) represent the mean as the mean plus/minus one standard deviation (in magenta color). A) Transverse relaxation rate constants R_2 determined for 4FdT4 applying diluted conditions. B) Transverse relaxation rate constants R_2 determined for 4FdT4 applying crowded conditions. The data have been acquired with a concentration of 4FdT4 of c^{4FdT4} = 50 µM at T = 298 K using B_0 = 18.8 T.

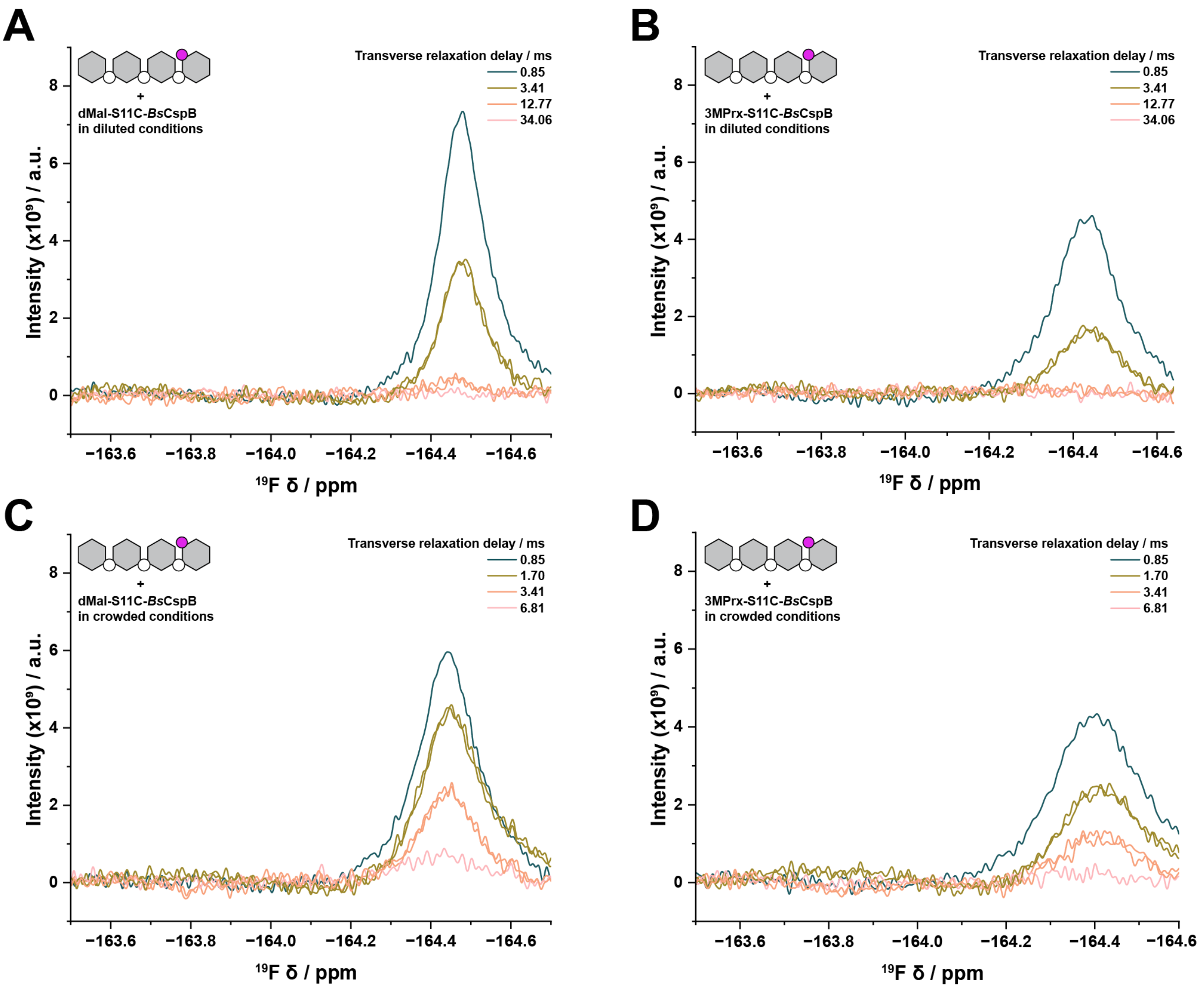

Figure S8. One-dimensional ^{19}F NMR spectra acquired for 4FdT4 in presence of either 3MPrx-S11C-*Bs*CspB or dMal-S11C-*Bs*CspB and varying transverse relaxation delays as well as in dependence on experimental conditions. A) 4FdT4 in complex with dMal-S11C-*Bs*CspB applying diluted conditions. B) 4FdT4 in complex with 3MPrx-S11C-*Bs*CspB applying diluted conditions. C) 4FdT4 in complex with dMal-S11C-*Bs*CspB applying crowded conditions. D) 4FdT4 in complex with 3MPrx-S11C-*Bs*CspB applying crowded conditions. The data have been acquired at T = 298 K using B_0 = 18.8 T. The upfield spectral range is limited due to a fluorine-containing paste in the probe head. This issue does not permit a reliable measurement of a broader upfield spectral range due to pronounced baseline rolling.

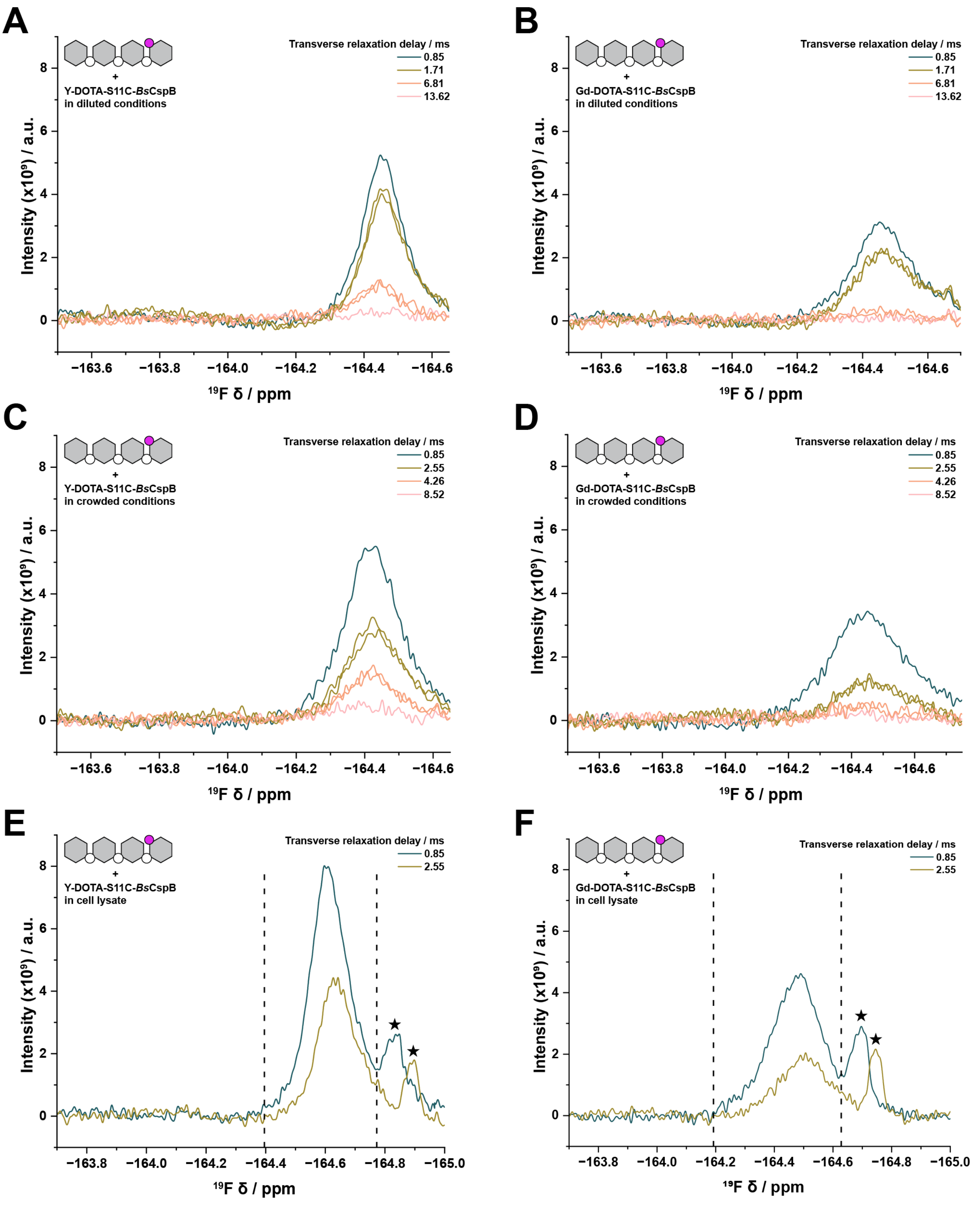

Figure S9. One-dimensional ^{19}F NMR spectra acquired for 4FdT4 in presence of either Gd-DOTA-S11C-*Bs*CspB or Y-DOTA-S11C-*Bs*CspB and varying transverse relaxation delays as well as in dependence on experimental conditions. A) 4FdT4 in complex with Y-DOTA-S11C-*Bs*CspB applying diluted conditions. B) 4FdT4 in complex with Gd-DOTA-S11C-*Bs*CspB applying diluted conditions. C) 4FdT4 in complex with Y-DOTA-S11C-*Bs*CspB applying crowded conditions. D) 4FdT4 in complex with Gd-DOTA-S11C-*Bs*CspB applying crowded conditions. E) 4FdT4 in complex with Y-DOTA-S11C-*Bs*CspB present in cell lysate. The vertical lines (dashed mode) represent the upfield and downfield limit, respectively, used to determine the two integrals. The resonance signals indicated using a star (in black) likely originate from the degradation of 4FdT4 under these conditions (see also Figure S6). F) 4FdT4 in complex with Gd-DOTA-S11C-*Bs*CspB present in cell lysate. The vertical lines (dashed mode) represent the upfield and downfield limit, respectively, used to determine the two integrals. The resonance signals indicated using a star (in black) likely originate from the degradation of 4FdT4 under these conditions (see also Figure S6). The data have been acquired at T = 298 K using B_0 = 18.8 T. Please note that the upfield spectral range is limited due to a fluorine-containing paste in the probe head. This issue does not permit a reliable measurement of a broader upfield spectral range due to pronounced baseline rolling.

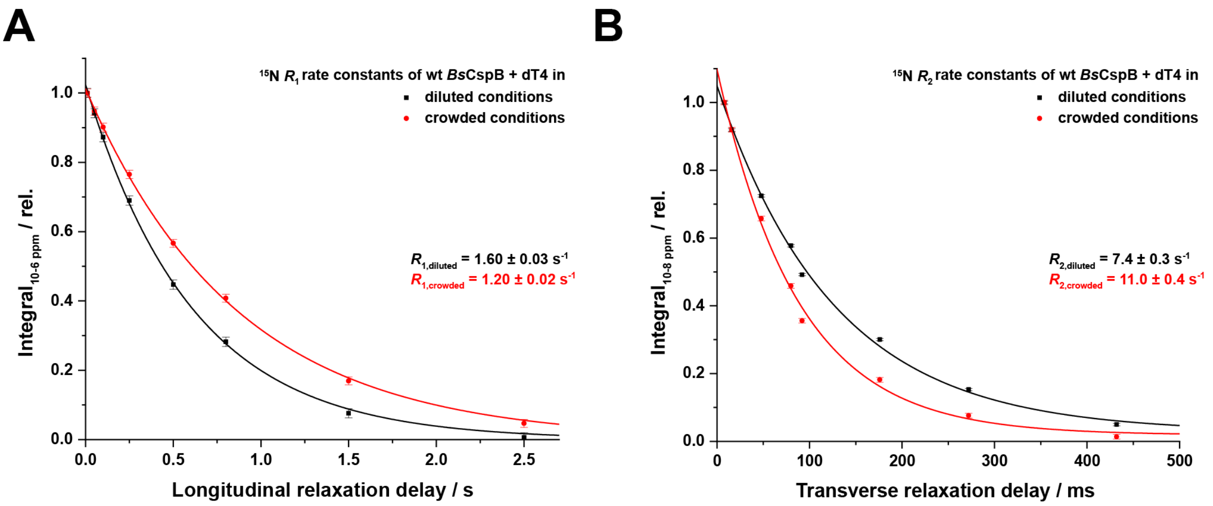

Figure S10. Determination of ^{15}N longitudinal and transverse relaxation rate constants R_1 and R_2 probing amide protons comprising *Bs*CspB in presence of dT4. Relative integrals determined for the spectral range between 10 and 6 ppm (R_1 measurements) and for the spectral range between 10 and 8 ppm (R_2 measurements) acquiring ^{15}N-edited ^{1}H NMR spectra for different relaxation delays. R_1 is shown in A) and R_2 is shown in B). Data acquired for diluted conditions are shown in black, data acquired for crowded conditions are shown in red.

References

[1] R. Sachs, K. E. Max, U. Heinemann, J. Balbach, *Rna* **2012**, *18*, 65-76.
[2] H. Schindelin, M. A. Marahiel, U. Heinemann, *Nature* **1993**, *364*, 164-168.
[3] T. Schindler, M. Herrler, M. A. Marahiel, F. X. Schmid, *Nat. Struct. Biol.* **1995**, *2*, 663-673.
[4] B. Köhn, M. Kovermann, *ChemBioChem* **2019**, *20*, 759-763.
[5] M. Zeeb, K. E. A. Max, U. Weininger, C. Löw, H. Sticht, J. Balbach, *Nucleic Acids Res.* **2006**, *34*, 4561-4571.
[6] F. Delaglio, S. Grzesiek, G. W. Vuister, G. Zhu, J. Pfeifer, A. Bax, *J. Biomol. NMR* **1995**, *6*, 277-293.
[7] B. A. Johnson, R. A. Blevins, *J. Biomol. NMR* **1994**, *4*, 603-614.
[8] J. Iwahara, C. Tang, G. Marius Clore, *J. Magn. Reson.* **2007**, *184*, 185-195.
[9] A. M. Mandel, M. Akke, A. G. Palmer, 3rd, *J. Mol. Biol.* **1995**, *246*, 144-163.
[10] A. G. Palmer, III, M. Rance, P. E. Wright, *J. Am. Chem. Soc.* **1991**, *113*, 4371-4380.
[11] H. Welte, T. Zhou, X. Mihajlenko, O. Mayans, M. Kovermann, *Sci. Rep.* **2020**, *10*, 1-12.
